# Mapping the genetic landscape of microbial lipid transformation in the intestinal microbiome

**DOI:** 10.1080/19490976.2026.2756735

**Published:** 2026-11-11

**Authors:** Laura C. Valk, Renate A. A. A. Ruigrok, Harro Timmerman, Johannes R. Björk, Qiyan Li, Shuyan Zhang, Rui Meneses, Jorge Francisco Vázquez Castellanos, Nicole de Wit, Jeroen Raes, Rinse K. Weersma, Michiel Kleerebezem, Arnau Vich-Vila, Jos Boekhorst

**Affiliations:** a Food and Health Group, Wageningen Food and Biobased Research, Wageningen University and Research, Wageningen, The Netherlands; b Department of Gastroenterology and Hepatology, University of Groningen, University Medical Center Groningen, Groningen, The Netherlands; c Department of Genetics, University of Groningen, University Medical Center Groningen, Groningen, The Netherlands; d Laboratory of Molecular Bacteriology, Department of Microbiology and Immunology, Rega Institute, KU Leuven, Leuven, Belgium; e VIB Centre for Microbiology, VIB, Leuven, Belgium; f Host-Microbe Interactomics Group, Animal Sciences Department, Wageningen University & Research, Wageningen, The Netherlands

**Keywords:** Small intestine, gut microbiome, lipid conversions, microbial metabolism, host-microbiome interaction

## Abstract

Dietary lipids are a key component in our diet, and fats and their derivatives have been associated with both beneficial as well as adverse effects on host health. However, the contribution of the gut microbiome to lipid metabolism remains understudied. Recognizing the importance of this interplay, we conducted extensive literature reviews to identify key bacterial genes involved in lipid metabolism, and created 88 Hidden Markov Models to allow rapid and accurate identification of these genes in genome and metagenome data. We applied these models to over 1000 metagenomes representative of the small intestine and colon, from both population-based and disease cohorts (LLDeep, MetaCardis and 1000IBD) to determine their lipolytic capacity. We observed that microbiome community types (enterotypes) carry different lipolytic potential. Serum levels of conjugated and sulfonated secondary bile acids were significantly associated with increased abundance of sterol-sulfonation genes (*cysC, cysD* and *BtSULT*). Small-intestinal communities were enriched in lipid genes compared to the colonic microbiota, underscoring the importance of microbial lipid metabolism in the small intestine compared to the colon. Overall, we demonstrate the value of our lipid metabolism models for functional microbiome profiling. Additionally, our study suggests that microbial activity in the upper gut might play a direct role in shaping host lipid metabolism, with potential implications for cardiometabolic health.

## Introduction

### The effect of the microbiome on dietary lipids is understudied

The important role of the gut microbiome in mediating the effects of diet on host health is well established.^1^ However, scientific efforts have been biased towards carbohydrate and protein metabolism, to date. This emphasis is reflected in one of the most widely used algorithms and frameworks for annotating metabolic functions in metagenomic data, the human gut metabolic modules (GMMs).^2^ Of the 103 modules (version 1.07), more than half are dedicated to saccharolytic functions, 39 modules are dedicated to proteolytic functions and only 11 modules are dedicated to lipolytic or lipid-associated functions. Although several tools exist to comprehensively profile carbohydrate metabolism in the gut microbiome, the community's capacity for lipid metabolism remains largely uncharacterized.^3^ ^,^ ^4^ The underrepresentation of lipid metabolism is significant given the important role lipids play in human health.^5^

Dietary lipids are predominantly triglycerides (TAGs), accounting for 90% of our total lipid intake. TAGs are composed of a glycerol backbone esterified to three long chain fatty acids (LCFA). The remaining dietary lipids consist of sterols, phospholipids, glycerolipids and fat-soluble vitamins.^6^ A small fraction of phospholipids has a hydrophilic residue (e.g., choline) attached to their glycerol backbone, while sterols represent the smallest lipid fraction^7^ Fats and their derivatives have been associated with both beneficial and adverse effects on host health, and current dietary recommendations state that up to 40% of our daily energy intake should be derived from beneficial fatty acids.^6^ ^,^ ^7^ Lipids with beneficial health effects are mostly TAGs containing unsaturated fatty acids, such as linoleic acid, and have been linked to improved cardiovascular health and inflammation reduction.^8-11^ Conversely, certain lipid classes, including saturated fatty acids, as well as lipid-derived compounds such as choline, have been implicated in negative effects on cardiovascular health and weight regulation.^12-14^

### Lipids and the small intestine

In the upper intestinal tract, host and bacterial derived lipases break down these complex dietary lipids into their constituent components; glycerol, free fatty acids and, when applicable, headgroups. Microorganisms possess the functional capacity to metabolize these breakdown products, rather than the intact lipid molecules.^15^ Recent studies have shown that changes in dietary lipid intake can rapidly and profoundly reshape the composition and metabolic activity of the gut microbiota, especially in the small intestine, which in turn influences host lipid digestion and absorption.^16-19^ However, the underlying mechanisms and implications of this change are not yet fully understood.^17^ ^,^ ^18^ One potential mechanism influencing the microbiome composition is the capacity of species to metabolize the digested dietary lipids. This is especially relevant for the species in the small intestine as, under normal conditions, 95% of the lipids are absorbed in the jejunum and do not reach the colon.^6^

Here we aimed to investigate the importance of the gut microbiota in the lipid metabolism, by determining the lipolytic capacity of bacterial communities in the small and large intestine and its associations with host health. We conducted an extensive literature study to identify all relevant genes associated with dietary lipid metabolization and constructed a comprehensive, manually curated *in silico* model for lipid metabolism, encompassing key bacterial genes for lipid component degradation, transformation and transport of lipids and their constituents. We employed a Hidden Markov Model (HMM)-based strategy to identify lipid metabolism-related genes of interest in (meta)genomes. This model was applied to assess the gut microbiome's potential to metabolize dietary lipid components in more than 1000 metagenomes, representative of either the small intestine or colon microbiomes from population-based and disease cohorts (LifeLines Deep,^20^ MetaCardis^21^ and 1000IBD^22^). In the fecal samples from the MetaCardis cohort, enterotype emerged as the strongest explanatory factor for variation in lipid-related gene content, and correlation analyses revealed associations between microbial lipid genes and serum lipid–associated metabolites. In contrast, no significant associations were detected in the population-based LifeLines Deep cohort. Notably, comparison of the LifeLines Deep cohort with the small intestinal dataset indicated that the small intestinal microbiome is more specialized for dietary lipid utilization. Collectively, these findings support a role for gut microbes mediating the effects of dietary lipids on host (metabolic) health and highlight the importance of incorporating the small intestinal microbiota to elucidate the underlying mechanisms.

## Results

### Definition of lipid genes and HMM development

Based on an extensive literature review, we created and curated a comprehensive set of Hidden Markov Models (HMMs) specifically designed to detect microbial genes within metagenomes which contribute to the metabolic capacity for dietary-lipid conversions (Supplemental Table S1). Experimentally validated gene genes were selected as seed sequences for the HMM construction. Reference organisms included both known inhabitants of the gastrointestinal (GI) tract as well as species associated with lipid metabolization isolated from other niches (Supplemental Table S2),^23^ enabling the detection of both established and potentially overlooked lipid conversions genes in gut microbial communities.

For clarity, these predicted lipid-related protein functions are hereafter referred to as lipid genes and refer to genes with direct or accessory functions in lipid metabolism. The HMMs covered both lipid-specific genes (involved exclusively in lipid metabolism or conversion) and accessory genes (associated with, but not limited to, the conversion of lipids and their derivatives). The lipid-specific transformations were subdivided into fatty acid transformation (with a focus on linoleic acid), β-oxidation, sterol transformation and degradation, choline degradation, sphingolipid and phospholipid modification, and membrane transportation. The accessory categories were added as TAG degradation produces glycerol^24^ ^,^ ^25^ and long chain fatty acids. Both glycerol and fatty acids are highly reduced substrates, and their oxidation generates reducing equivalents that must be transferred to electron acceptors to maintain cellular redox balance.^26^ ^,^ ^27^ Consequently, we included genes involved in electron transfer and energy conservation (e.g., nuo and nqr) as well as glycerol conversion and degradation as accessory functions that may support the utilization of lipid-derived substrates, while acknowledging that these genes are not specific to lipid metabolism. Additionally, we incorporated key genes for the synthesis of vitamin K and B2, as these two vitamins have been associated with lipids.^28^ ^,^ ^29^ In total, we identified 88 key genes (Supplemental Table S1) associated with lipid transformation.

To evaluate the functional specificity of the resulting HMMs, all seed proteins were analyzed with InterPro^30^ (Supplemental Table S3, Figure S1). Functional predictions from the integrated NCBIfam, Pfam, PANTHER, HAMAP, and other InterPro member databases were highly consistent with the expected functions of the experimentally validated seed proteins. Most seed proteins received enzyme-level functional assignments, while the remainder were supported by congruent protein family- or domain-level annotations. Only one HMM (nuo_J11) lacked a corresponding functional prediction in the queried databases. Together, these results provide strong support for the functional specificity of the developed HMM collection.

### Lipid gene distribution in the unified human gastrointestinal genome (UHGG) database

To investigate the distribution and co-occurrence of lipid metabolism genes in gut-associated microorganisms, we analyzed 289,232 publicly available genomes from the UHGG database^31^ (v 2.0.2) for genetic potential related to lipid metabolism. *Escherichia coli* and *Klebsiella pneumoniae* exhibited the largest lipid-metabolism gene repertoires, with 74 and 71 distinct genes detected at the species level, respectively, and up to 64 and 65 genes detected in individual genomes, respectively. The 88 lipid-associated genes were broadly distributed across 92 orders and 3411 families (Figure 1, Supplemental Figure S2). Despite their widespread occurrence, the diversity of lipid genes varied substantially among taxa. Members of the order *Enterobacterales* harbored the highest number of lipid genes per genome, indicating a potential for a broad range of both direct and accessory lipid metabolic functions. In contrast, genomes belonging to *Lactobacillales* harbored a more restricted set of lipid genes but uniquely encoded linoleic acid metabolism genes, including *CLA-DH* and *CLA-DC*.

To assess whether lipid metabolic potential reflects phylogenetic relationships, we analyzed a subset of 4640 representative genomes, comprising one genome per species. A moderate correlation was demonstrated between functional and phylogenetic distances with a Mantel test (r = 0.56, *p* = 0.001, 1000 permutations), indicating that closely related species tend to possess more similar lipid gene repertoires.

The prevalence of individual lipid genes varied considerably across genomes (Figure 1). Across the 974 bacterial species, the prevalence of lipid genes varied widely. The most prevalent genes were *tpiA* (96.4%), *dhaT* (78.1%), *glpK* (70.2%), *ribE* (67.8%), *mdh* (66.0%), *fadH* (65.1%), *fakA* (62.2%), *cutD* (56.1%) and *CLA-HY* (53.5%) (Supplemental Figure S2). Among these highly prevalent genes, core representation ranged from 30.6% to 51.4%, with *dhaT* showing the highest proportion of core representation (51.4%), followed by *glpK* (46.6%) and *fakA* (46.4%).

In contrast, *eRG3*, *cyp125* and *choD* were detected in only 1 species (Figure 1, Supplemental Figure S2). Patterns of gene co-occurrence likewise differed substantially among lipid metabolic pathways (Figure 1, Supplemental Figure S2, Supplemental Table S4). Genes associated with the electron transport chain (nuo and nqr clusters) and choline transformation (cutC and cutD) formed distinct co-occurrence clusters, whereas genes associated with β-oxidation (the fad cluster) were more broadly dispersed across genomes. These observations suggest that lipid metabolism genes do not consistently cluster into discrete pathways and that many may have functions extending beyond lipid metabolism. Nevertheless, this analysis provides a comprehensive reference framework of the lipid metabolic potential encoded by gut-associated microorganisms.

To determine how this repertoire is represented at the community level, we next examined the prevalence and distribution of microbial lipid genes in metagenomes from gastrointestinal environments, where microbial communities could contribute to dietary lipid metabolism.^16-19^

**Figure 1.**
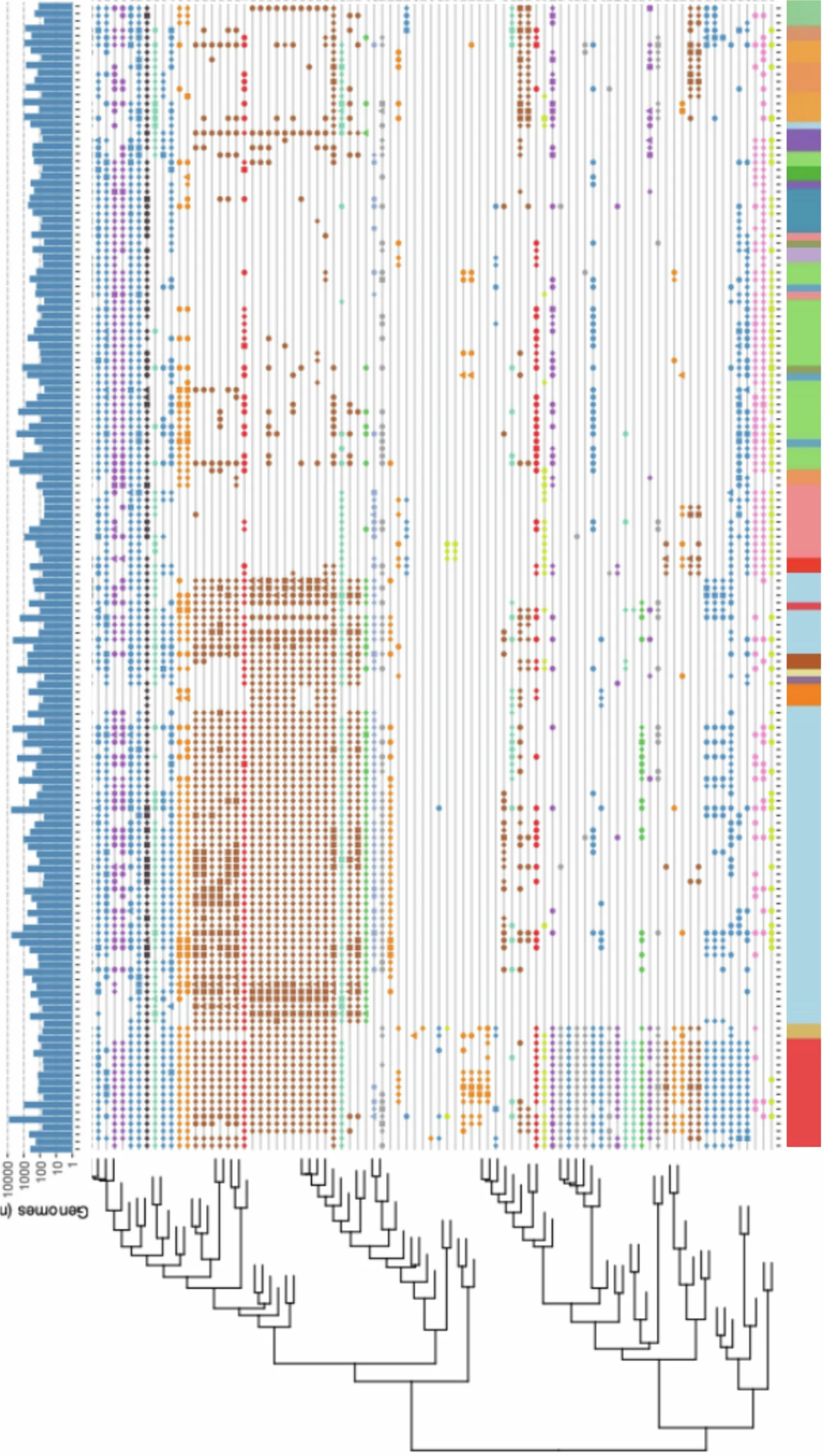
Distribution of the lipid genes across genomes of the unified human gastrointestinal genome (UHGG) database. All genomes in the UHGG database were analyzed for their genetic lipid metabolization potential with the lipid gene dataset. Genomes containing lipid gene with a prevalence of at least 1% across their order were visualized. Lipid genes were hierarchically clustered based on their co-occurrence patterns using Euclidean distanced and complete linkage hierarchical clustering. Genes are colored according to their functional group.

### Lipid gene assessment in relation to enterotypes and serum metabolites using the MetaCardis BMIS cohort data

To evaluate the relationship between lipid-metabolizing genes and host health parameters, we applied our curated HMM set to the fecal samples of the MetaCardis sub-cohort, BMIS.^21^ This cohort was selected due to its inclusion of both healthy individuals and participants diagnosed with pre-diabetes or metabolic syndrome, providing a relevant context for studying microbial lipid metabolism. In the original characterization of this cohort, gut community typing identified four enterotypes, (*Bacteroides*1 (Bact1), *Bacteroides*2 (Bact2), *Prevotella* (Prev), and *Ruminococcus* (Rum)), with the Bact2 enterotype in particular found to represent a low-cell-density, dysbiotic configuration whose prevalence increased with BMI and was associated with elevated systemic inflammation (hsCRP), dysmetabolism, and ischemic heart disease.^21^

Notably, analysis of the genetic potential for lipid metabolism across samples revealed that enterotype explained the largest proportion of variation (13%) in the abundance of lipid-metabolizing genes (Supplemental Figure S3, Supplemental Table S5), compared to other environmental and host-related factors.^21^ Of the 88 lipid genes, 81 were detected across the cohort (Supplementary Table S6), with up to 78 unique lipid genes observed within a single metagenome. Participants with a *Prevotella* enterotype exhibited a significantly lower lipid gene richness (Wilcoxon rank-sum test, mean = 51.08 ± 6.1, *p* < 0.05) (Figure 2A, Supplemental Table S7). In contrast *Bacteroides* 2 had a significantly higher ratio of unique lipid genes relative to the total number of unique genes (mean = 0.018 ± 0.007%, *p* < 0.05) (Figure 2B, Supplemental Table S7). Because *Bacteroides 2* enterotype is known to have the lowest microbial load,^32^ a factor that could increase sequencing depth and thereby inflate estimates of gene richness, we examined sequencing depth across enterotypes and evaluated potential correlations between sequencing depth and the number of unique lipid genes detected. Although metagenomes from the *Bacteroides 2* enterotype indeed exhibited the lowest microbial load and the highest sequencing depth (mean: Bact2 = 2.9 * 10^−4^, Bact1 = 1.9 * 10^−4^, Rum = 2.0 * 10^−4^, Prev = 2.2 * 10^−4^, Supplemental Figures S4A), we did not observe a significant correlation between sequencing depth and the total number of unique lipid functions across samples (r = 0.015, *p* = 0.7, Supplemental Figure S4B). These findings indicate that the higher richness of lipid genes observed in *Bacteroides* 2 reflects differences in genetic lipid-metabolizing potential rather than an artifact of sequencing depth.

**Figure 2.**
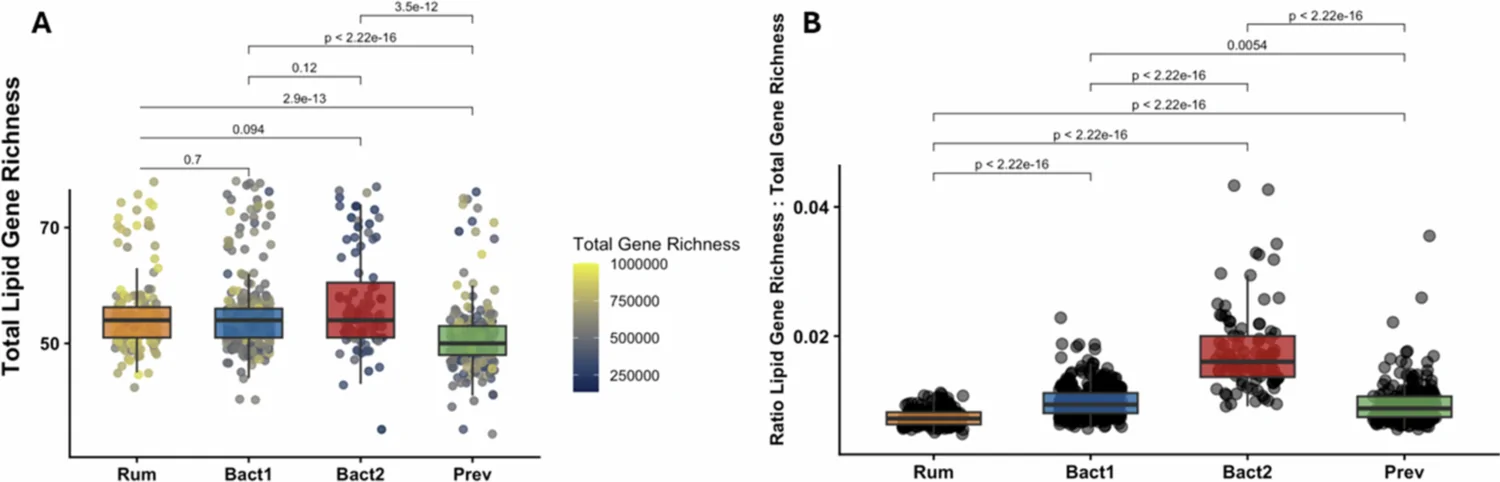
Variation in unique microbial lipid genes across enterotypes in the MetaCardis-BMIS cohort. (A) Total lipid gene richness per metagenome across enterotypes. Dots are colored according to number of unique gene sequences (total gene richness), with yellow indicating high richness and dark blue indicating low richness. (B) lipid gene richness relative to the total gene richness^21^ categorized according to enterotype. In both panels, the central boxes represent the interquartile range (IQR), spanning from the first quartile (Q1) to the third quartile (Q3). The horizontal line within the box indicates the median (Q2). Whiskers extend from the box to the smallest and largest values within 1.5 times the IQR from the lower and upper quartiles, respectively. Data points are plotted individually. Statistical significance between enterotypes was assessed using the Wilcoxon rank-sum test.

Principal Coordinate Analysis (PCoA) based on Bray–Curtis dissimilarity of lipid gene abundance profiles revealed statistically significant variation in lipid metabolism potential across samples and enterotypes (pairwise PERMANOVA; R^2^: 0.05–0.23) (Figure 3, Supplemental Table S8). Notably, the *Prevotella* enterotype exhibited the most distinct and highest dispersion of lipid metabolization profile among the four enterotypes. This separation was predominantly driven by the prevalence of accessory genes (vitamin K and electron transport chain) coupled with the reduced abundance of lipid genes associated with glycerol degradation, β-oxidation and sterol transformation. The largest separation was between enterotypes *Ruminococcus* and *Bacteroides 2* (pairwise PERMANOVA; FDR = 0.001; R^2^ = 0.23). In contrast, samples classified as *Ruminococcus* enterotype exhibited the largest overlap with *Bacteroides 1* enterotype in the PCoA analysis (pairwise PERMANOVA; FDR = 0.001; R^2^ = 0.05) (Figure 3, Supplemental Table S8).

**Figure 3.**
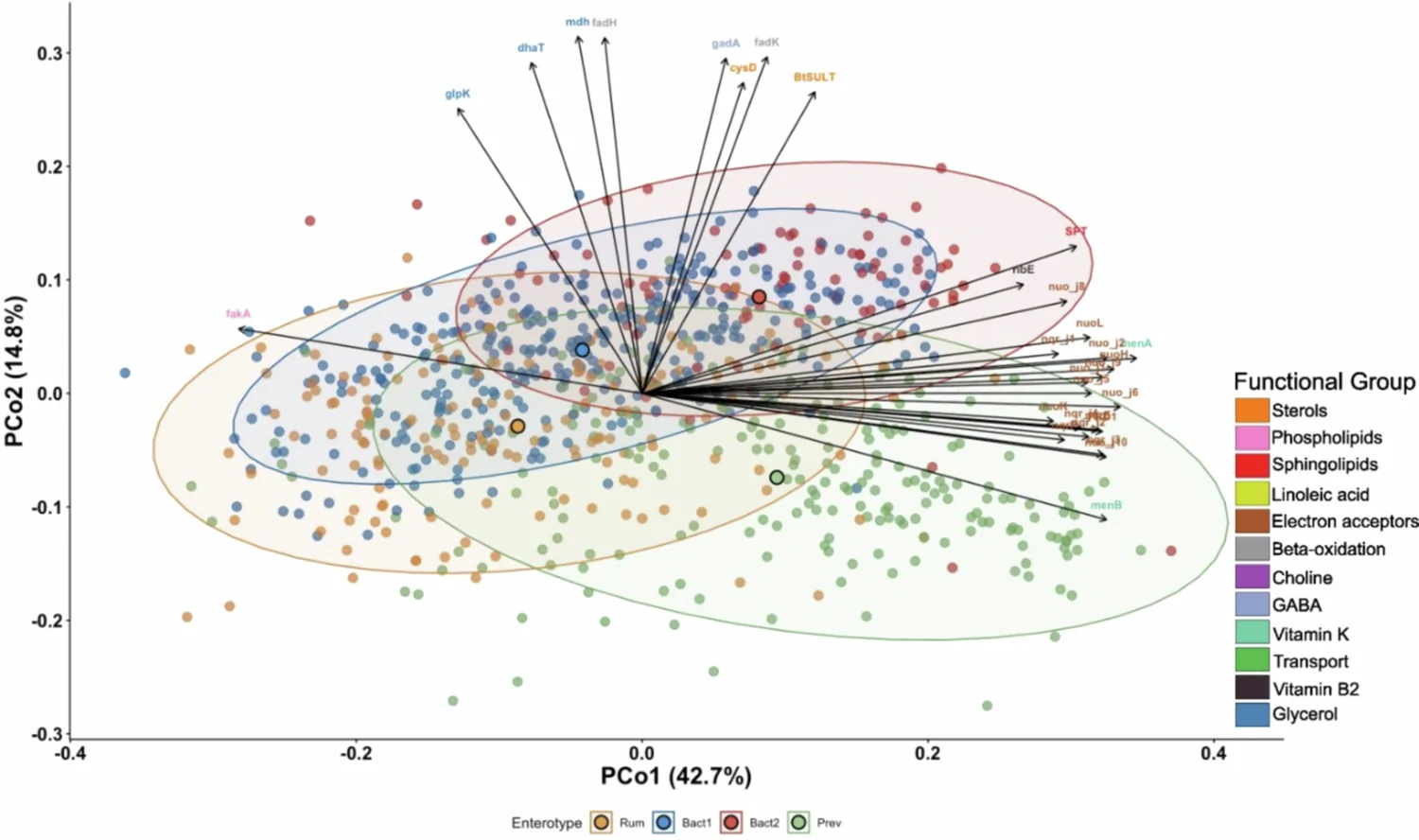
Distinct enterotype clustering driven by lipid gene abundance (MetaCardis-BMIS). Principal coordinate analysis based on Bray–Curtis distances calculated from the relative abundances of lipid genes in the MetaCardis-BMIS cohort. Each dot represents one sample (metagenome), colored by enterotype: yellow for *Ruminococcus* (Rum), blue for *Bacteroides* 1 (Bac 1), red for *Bacteroides* 2 (Bac 2) and green for *Prevotella* (Prev). Arrows indicate the 30 lipid genes with the highest loadings, showing their contribution to variation along the first two PCo axes, ellipses are 2D approximations at a confidence level of 90% and the outlined circles are the centroids of the ellipses. Lipid genes are colored according to their functional group.

To assess whether individual lipid functions exhibited distinct patterns across enterotypes, we performed pairwise comparisons of lipid gene abundances between enterotypes (Figure 4A, Supplemental Table S9). For this analysis, lipid genes with a prevalence greater than 20% across the cohort (*n* = 62) were retained. Of the 62 genes, 60 differed significantly between enterotypes. The enterotypes displayed distinct lipid gene profiles. Because the majority of lipid genes differed significantly between enterotypes, we highlight genes belonging to biologically interpretable pathways or functional lipid group. Sterol-sulfonation genes (*cysC, cysD, btSULT*)^33^ were significantly enriched (FDR < 1.05 * 10^−9^) in *Bacteroides* 2 and, to a lesser extent, in *Bacteroides* 1 (FDR < 1.24 * 10^−13^). Choline transformation genes, *cutC* and *cutD*, which mediate the conversion of dietary choline to trimethylamine (TMA),^34^ were also most significantly enriched in *Bacteroides* 2 (FDR < 0.05). Genes associated with exogeneous fatty acid incorporation (*fakB2*, *fakA*)^35^ and the membrane bound fatty acid hydratase (*CLA-HY*)^36^ were significantly enriched in *Bacteroides* 1 and *Ruminococcus* but underrepresented in *Bacteroides* 2. Furthermore, electron acceptor genes (*nuo*-genes, *FDR1, FDR2*, *sdhA*, *hypS*, *frhD*) were consistently enriched in the *Prevotella* and *Bacteroides* 2 enterotypes (FDR < 0.05).

To explore potential functional implications, we conducted a hierarchical all-against-all correlation analysis between lipid gene and serum metabolite abundances.^37^ In total, 472 lipid metabolites were tested for associations with the lipid genes, yielding 228 significant associations between lipid metabolite clusters and gene clusters (FDR = 1.24 × 10^−8^ – 0.01, Figure 4B, Supplemental Figure S5, Supplemental Table S10). The strongest association was observed between *ribE* (vitamin B2 biosynthesis) and phenylacetyl carnitine (Spearman's− rho = –0.35; FDR = 1.24 × 10^−8^; Figure 4B). The second most significant signal was a positive association with a distinct set of genes (*cysC, cysD, BtSULT, dbhC, nuo_J11* and *fadK*) and multiple bile acid metabolites, including ursodeoxycholate (UDCA), iso-UDCA, glycol-UDCA, glycol-UDCA-sulfate, tauro-UDCA and tauro-UDCA-sulfate (Spearman's rho = 0.17-0.35; FDR = 1.24 × 10^−8^ − 0.025; Figure 4B, Supplemental Table S10). Notably, the genes *cysC*, *cysD* and *BtSULT* are part of a bacterial sulfotransferase pathway involved in the sulfonation of steroidal metabolites, including cholesterol and select bile acids, and have been linked to the production of sulfated steroid derivatives in the gut microbiome.^33^ In addition, all genes in this set were enriched in *Bacteroides* 2 enterotype relative to the other enterotypes (Figure 4A, Supplemental Table S9).

Interestingly, three genes (*fakB2, CLA-HY* and *fakA*) showed positive associations with numerous long-chain fatty acids. This may suggest that these genes, which are involved in exogenous fatty acid incorporation (*fakB2*, *fakA*)^35^ and the membrane bound fatty-acid hydratase (*CLA-HY*),^36^ act on fatty acids extracellularly. However, as these genes were positively associated with up to 88% of serum metabolites (Supplemental Figure S5, Supplemental Table S10), including many without an obvious mechanistic link to fatty acid metabolism, indicating that additional factors likely contribute to these associations.

**Figure 4.**
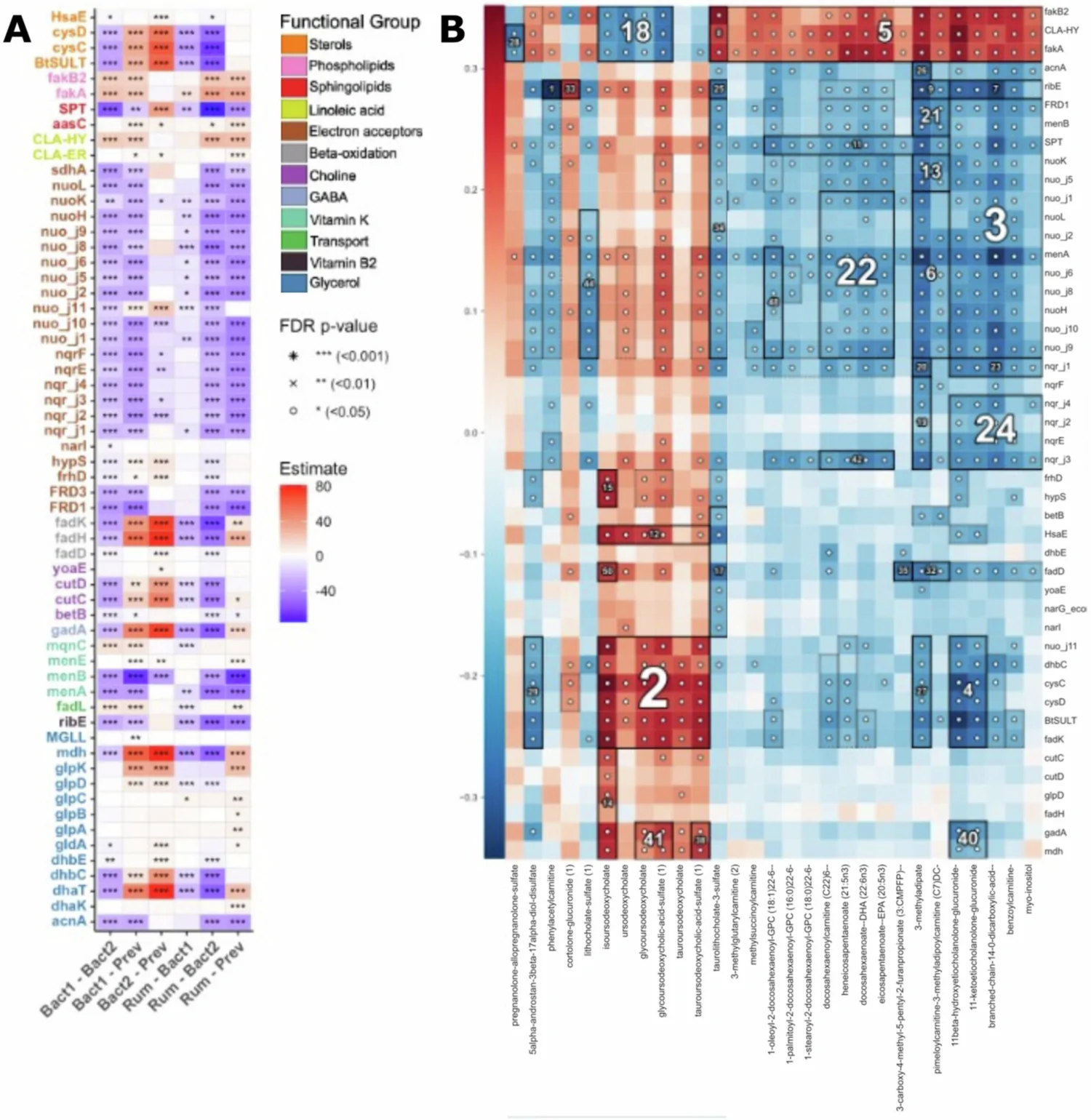
Lipid gene abundance differences between enterotypes and associations with serum lipid levels (MetaCardis-BMIS). (A) Differential abundance of lipid genes detected in >20% of samples between enterotypes, assessed using linear regression followed by Tukey's post-hoc test. Estimate refers to the model coefficient representing the difference in the outcome between enterotypes (e.g. *Bact1* relative to *Bact2*). Red indicates enrichment, blue underrepresentation. Significance: *FDR < 0.05, **FDR < 0.01, ***FDR < 0.001. (B) Hierarchical All-against-All association testing (HAllA)^37^ between microbial lipid gene abundances per metagenome and serum lipid metabolites (relative abundance). The plot shows a subset of the most significant associations, for the complete top 50 associations see Supplemental Figure S5. Red indicates positive associations and blue negative associations. Color intensity reflects the strength of the association (Spearman's rho).

To investigate the potential relationship between fecal lipid metabolization potential and metabolic health, we stratified samples according to participant health status (healthy control (CTRL), prediabetes (PD), metabolic syndrome without PD (MBS) and MBS with PD (MBS-PD). We compared the lipid richness and ratio of lipid richness to total gene richness across groups (Figure 5A, Supplemental Tables S11 and 12). No significant difference was observed in total unique lipid functions between the groups (mean: CTRL = 53, MBS = 54, PD and MBS/PD = 55; FDR < 0.05). However, the ratio of unique lipid functions normalized by the total gene richness per metagenome was significantly higher in both the metabolic syndrome (MBS, mean = 1.2 * 10^−2^, *p* < 0.01) and the combined group (MBS/PD, mean = 1.0 * 10^−2^, *p* < 0.01) compared to the control (mean = 0.9 * 10^−2^) (Figure 5B, Supplemental Table S12). The increased relative lipid gene richness observed in the MBS-associated groups appeared to be driven by the higher prevalence of the *Bacteroides* 2 enterotype within these individuals (Supplemental Figure S6). However, MBS status remained independently associated with lipid gene richness after correction for enterotype (FDR < 0.05; Supplemental Table S13). Pairwise comparison of the lipid gene abundance compared to the control group revealed that phospholipid-associated genes (*fakA, fakB2*) and fatty acid hydratase (*CLA-HY*) were significantly underrepresented in the MBS/PD group compared to controls (FDR < 0.05; Figure 5C, Supplemental Table S13). *fakB2* was also significantly underrepresented in the MBS group (FDR < 0.001).

**Figure 5.**
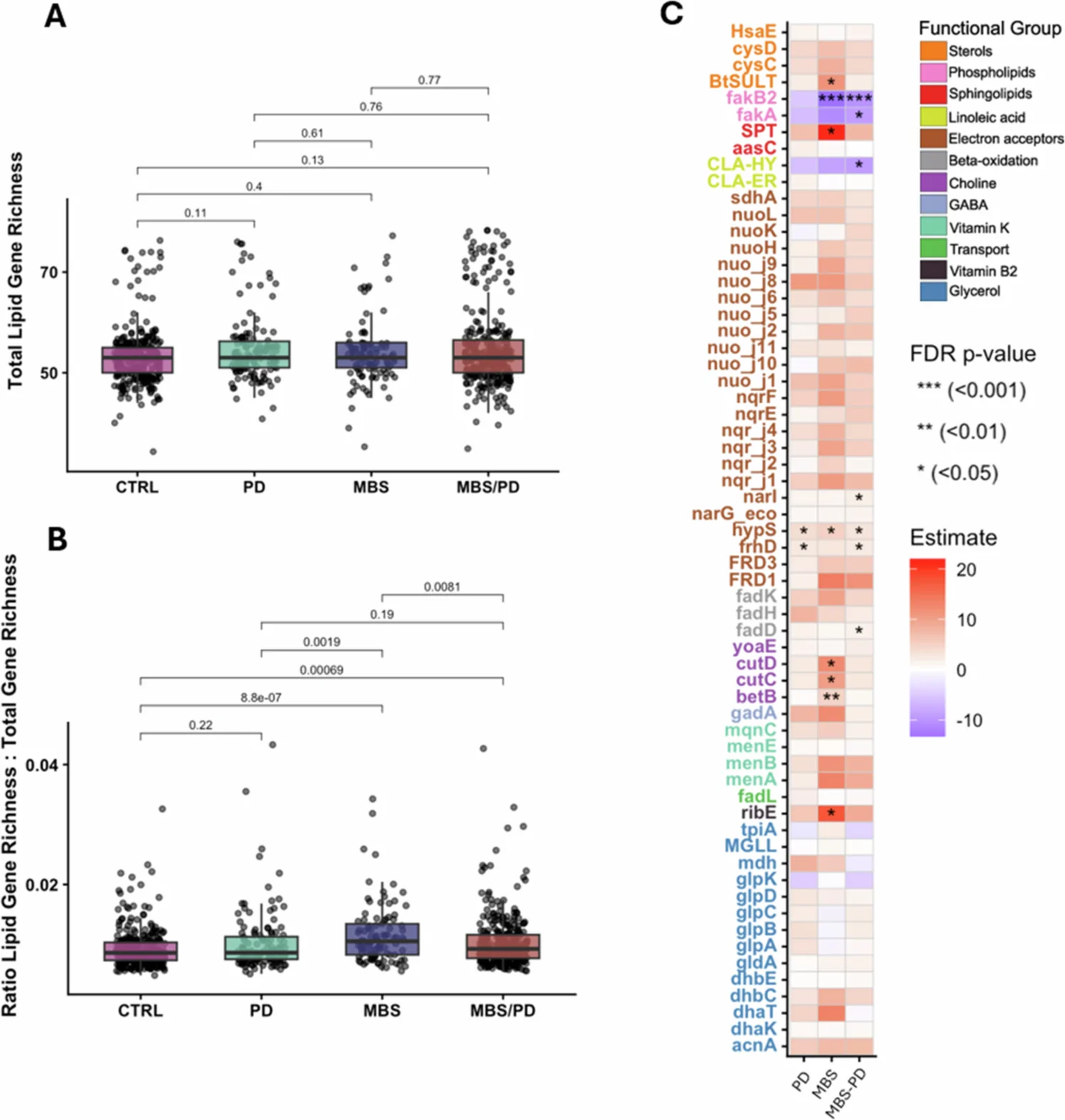
Lipid-metabolizing potential demonstrated limited variation across health status groups in the MetaCardis Cohort participants. (A) Total lipid gene richness and (B) lipid gene richness relative to total unique genes in the fecal communities of MetaCardis-BMIS participants, categorized according to participant metabolic health status. The central boxes represent the interquartile range (IQR), spanning from the first quartile (Q1) to the third quartile (Q3). The horizontal line within the box indicates the median (Q2). Whiskers extend from the box to the smallest and largest values within 1.5 times the IQR from the lower and upper quartiles, respectively. Data points are plotted individually. Statistical significance between the different groups was assessed using the Wilcoxon rank-sum test. (C) Pairwise comparison of relative lipid gene abundance between controls and individuals with prediabetes (PD), metabolic syndrome without PD (MBS), or metabolic syndrome with PD (MBS/PD). Red indicates enrichment and blue underrepresentation within the respective group versus control. Significance: *FDR < 0.05, **FDR < 0.01, ***FDR < 0.001. Lipid genes are colored according to their functional group.

### Lipid gene assessment in the general population (LLDeep)

To assess whether the lipid-metabolizing potential of the gut microbiome is associated with broader host health parameters within the general population, we analyzed a subset (*n* = 255) of the LifeLines DEEP (LLD) cohort,^20^ for which fecal metagenomic and metabolomic data were available. Hierarchical all-against-all clustering revealed identified 352 significant association (FDR < 0.01) between lipid genes and lipid-related fecal metabolites (Supplemental Figure S7, Supplemental Table S15). The strongest associations (FDR = 7.96 × 10^−12^) were with three lipid genes (*fadK, mqnC* and *nuo_J6*) and 16 lipid related metabolites. These metabolites encompassed lipid-related metabolites and derivatives, including dodecanedioate, (S)-3-hydroxybutyrylcarnitine, linoleoyl ethanolamide and 3-methyladipate. However, despite the statistical significance of these associations, no clear or direct functional relationships between specific genes and metabolites could be discerned, suggesting that these patterns may reflect indirect or system-level links rather than direct metabolic coupling.

We next explored whether host phenotypic factors could explain variability in lipid gene abundances. Differential abundance analyses using the diet score and the Gut Microbiome Wellness (GMW2) index^38^ and lipid gene abundances were conducted. While this analysis revealed no significant correlation with the diet score (Supplemental Figure S8A, Supplemental Table S16), we identified 12 significant associations between lipid gene abundances and the GMW2 Index (Supplemental Figure S8B, Supplemental Table S17). A distance-based redundancy analysis with host phenotype identified only ACE inhibitor use as significantly associated with individual lipid genes (Supplemental Figures S9, Supplemental Table S18) at an FDR threshold of 0.1 (FDR = 0.078), indicating the low explanatory contribution (R^2^ = 0.013) of host phenotype. In contrast, stronger associations observed in the disease-specific MetaCardis cohort suggest that such relationships become more apparent under perturbed metabolic conditions and/or in larger cohorts. In the context of lipid metabolism specifically, dietary lipids are predominantly absorbed in the small intestine and would be therefore expected to exert the strongest influence on the microbiome in that specific niche. Microbially mediated lipid-processing functions occurring upstream in the gastrointestinal tract are difficult to capture using fecal metagenomic profiling.^39^ Together with substantial inter-individual dietary variability and functional redundancy within the gut microbiome,^1^ ^,^ ^39^ ^,^ ^40^ this may explain the limited explanatory power observed in the general population.

We therefore next compared lipid gene repertoires between small intestinal microbiomes and fecal microbiomes from the LLD cohort to assess location-specific differences in lipid metabolism.

**Figure 6.**
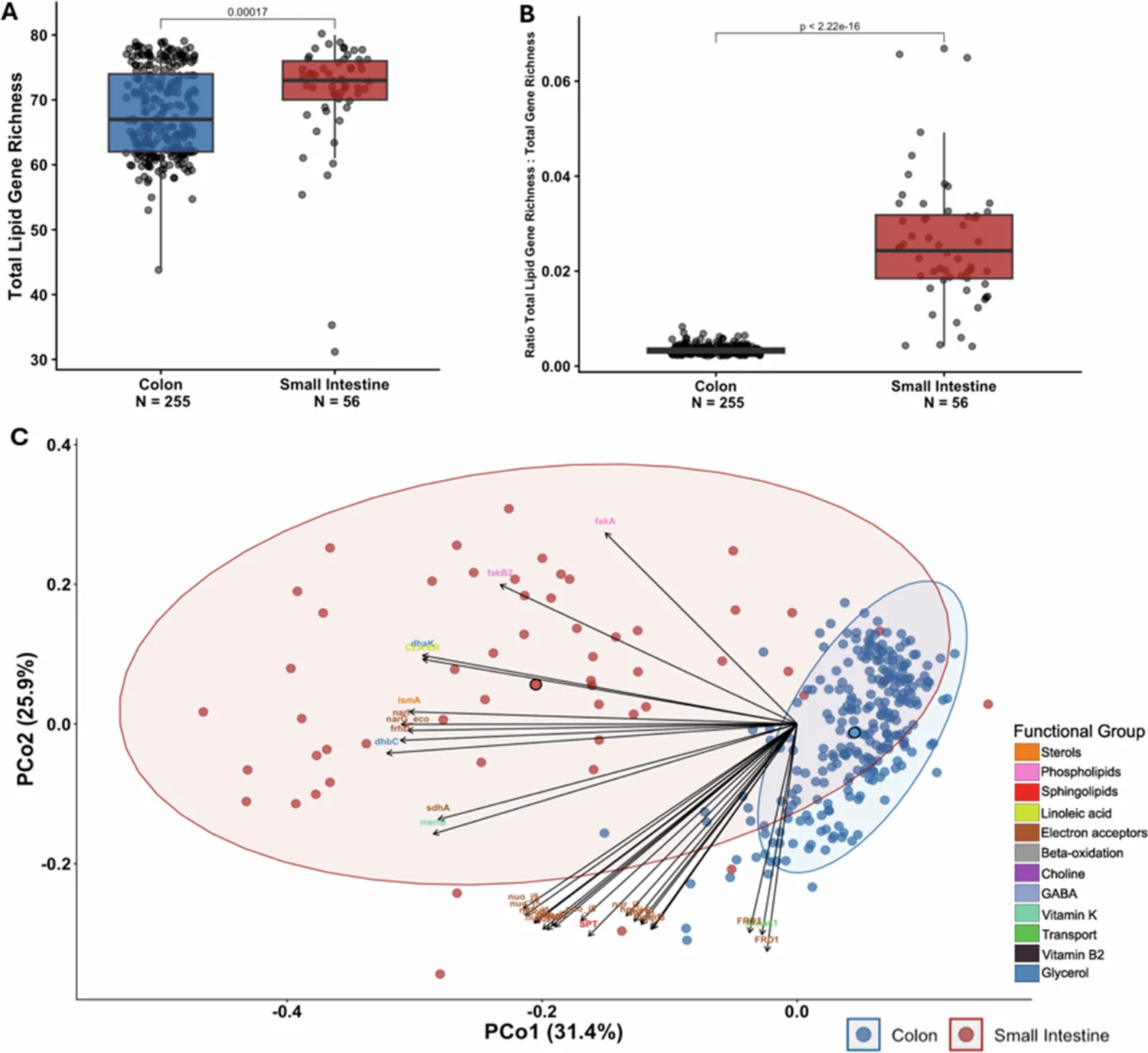
Increased lipid functional richness and variability in the small intestine versus colon. (A) Total lipid gene richness. Each dot represents the total number of unique lipid genes per sample. (B) ratio of unique lipid genes normalized by the total gene richness per sample in small intestinal (1000IBD) and colonic metagenomes (LLD). The central boxes represent the interquartile range (IQR), spanning from the first quartile (Q1) to the third quartile (Q3). The horizontal line within the box indicates the median (Q2). Whiskers extend from the box to the smallest and largest values within 1.5 times the IQR from the lower and upper quartiles, respectively. Data points are plotted individually. Statistical significance between the different groups was assessed using the Wilcoxon rank-sum test. (C) Principle coordinate analysis with as distance matric the Bray–Curtis distance of the abundance of lipid metabolization genes in the small intestine (red) and colon (blue) microbiomes. Dots represent one sample, arrows highlight the lipid genes most strongly correlated with the first two PCoA axes, ellipses are 2D approximations at a confidence level of 90% and the outlined circles are the centroids of the ellipses.

### Lipid gene assessment in the small intestine microbiome (1000IBD) compared to colonic microbiome (LLD)

Given the limited significant associations observed in fecal microbiomes (largely representative of the lower intestinal microbiome and therefore hereafter referred to as colonic microbiome) in a general population cohort, we next examined whether lipid-metabolizing potential differs in microbiomes from the small intestine, where lipid absorption primarily occurs. A subset (*n* = 56) of the 1000IBD cohort,^22^ including participants with an ileostomy (*n* = 47) or ileoanal pouch (*n* = 9) was selected as representative of small intestine microbiomes. Of this subset, eight participants were reported to have active disease, while the remainder were reported as healthy. Since the community composition of the active-disease participants did not differ from that of the healthy samples, they were included in the analysis. Comparison of the lipid metabolization potential of the colonic microbiomes of the LLD cohort and small intestine microbiomes revealed that the latter exhibited a higher lipid gene richness (mean = 71, *p* < 0.01, Figure 6A, Supplemental Table S19). When normalized by total gene richness per sample, this difference became even more pronounced, indicating that small intestinal communities allocate a larger proportion of their genetic capacity to lipid metabolism compared to colonic communities. (Figure 6B, Supplemental Table S19). Furthermore, small intestine microbiomes displayed more interindividual diversity in terms of lipid gene composition compared to the fecal microbiomes (Figure 6C, Supplemental Table S20), in line with the greater within-group microbial variability previously reported in the small intestine.^41^ ^,^ ^42^

We next compared the abundance of the small lipid gene in the colon compared with the small intestine. Among the lipid genes with at least 20% prevalence (*n* = 78), 56 were significantly more abundant in the small intestine (Figure 7A, Supplemental Table S21). Only the choline transformation genes (*cysC, cysD*), glycerol kinase (*glpK*), dehypoxanthine futalosine cyclase (*mqnC*) and 2,4-dienoyl-CoA reductase (*fadH*) were more abundant in colonic microbiomes. This observation underscores that small intestine communities have both greater diversity and higher overall abundance of lipid genes compared to colon-associated communities.

**Figure 7.**
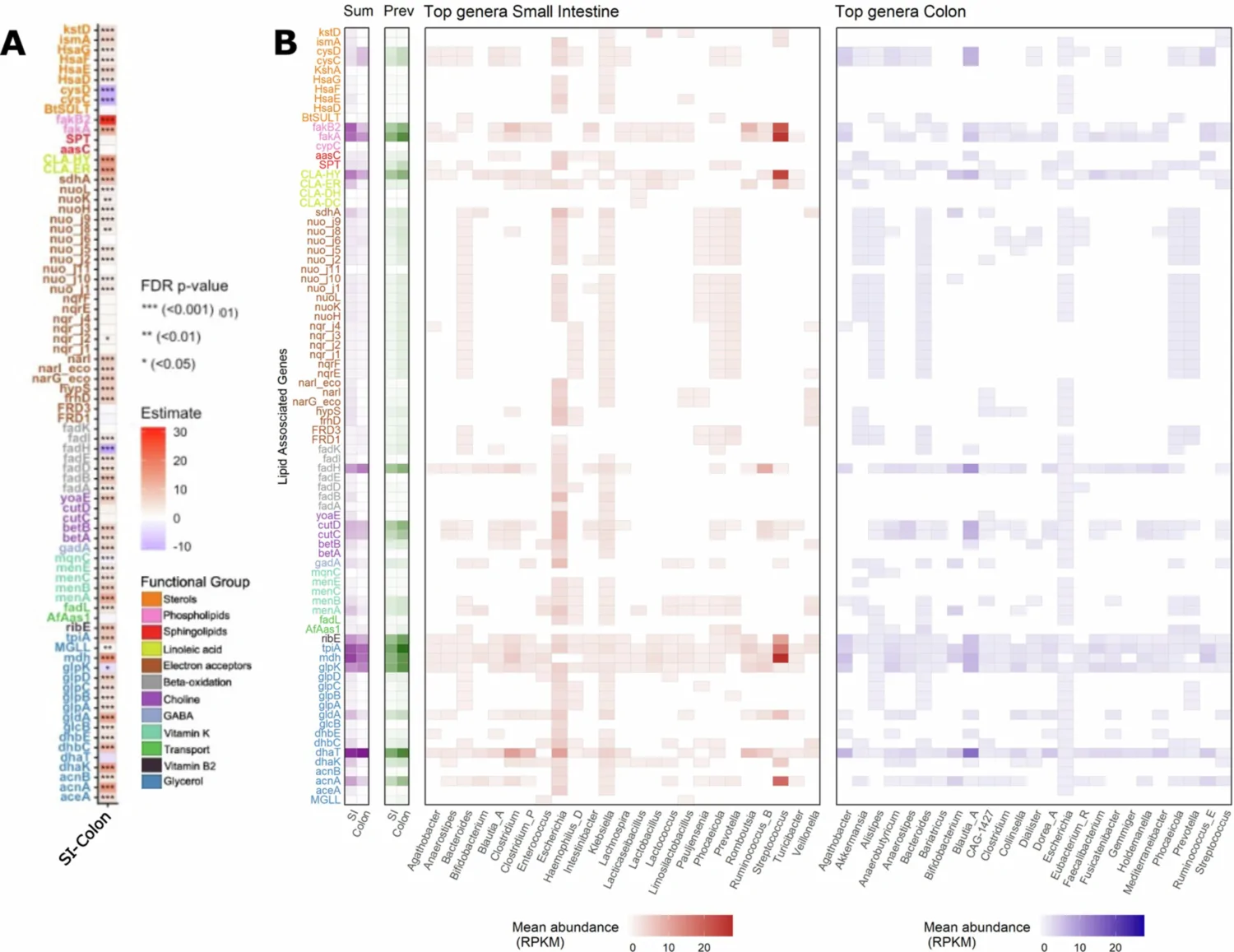
Pairwise comparison of lipid gene relative abundances between small intestine and colon microbiomes. (A) Lipid genes with a prevalence of more than 20% in the LLD (colon) and 1000IBD (small intestine) cohorts combined. Associations were corrected for participant age (at sampling), sex and body mass index (BMI). Estimate refers to the model coefficient representing the difference in outcome between colon and small intestine samples (small intestine relative to colon). Significance: *FDR < 0.05, **FDR < 0.01, ***FDR < 0.001. Lipid genes are colored according to their functional group. (B) Heatmaps display (from left to right) the sum and prevalence of mean gene abundance (RPKM) and the top 25 contributing taxa per environment for lipid metabolism genes identified in the respective microbiomes. In the color scale white indicates low abundance, and dark colors indicate high abundance. Lipid genes are colored according to their functional group.

To identify which genera carry the genes influencing dietary lipid metabolism in each environment, we analyzed genus-level lipid gene abundance in both intestinal environments (Figure 7B, Supplemental Table S22). Two distinct patterns were observed among lipid genes that were substantially more abundant in the small intestine relative to colonic communities. First, for highly abundant genes, this enrichment was primarily driven by their high prevalence or by their association with the highly abundant *Streptococcus* genus (e.g., *acnA* and *mdh*). Second, for other genes, the enrichment in the small intestine resulted from their near absence in the colon (e.g., *betA* and *betB*). Although the per-gene abundance was higher in the small intestine in 67 out of 78 genes, the majority of the genes (74 out of 78) were more prevalent in the colon as a larger number of low-abundance genera carried lipid genes (Figure 7B, Supplemental Table S22).

*Klebsiella* was the genus with the highest number of lipid genes, totaling 64 unique genes (Figure 7B, Supplemental Table S22). Interestingly, another genus from the same taxonomic order, *Escherichia*, ranked second with 61 unique lipid genes. While a substantial portion (40 and 38, respectively) of these counts was due to accessory genes, such as those encoding components of the electron transport chain (*nuo* and *nqr*-genes), both genera also harbored numerous dedicated lipid functions, for example, genes involved in sterol degradation (*KstD* and *HsaDEFG*) and β-oxidation (*fad*-genes). This pattern reflects the broad metabolic flexibility previously attributed to this order.^43^

## Discussion

Our modelling framework captures a broader repertoire of lipid‑associated functions, including 42 genes involved in the transformation of lipid‑derived compounds such as fatty acids and choline, thereby providing a more comprehensive representation of the dietary lipid‑modifying potential of the gut microbiome. It encompasses lipid transformations with potential health implications, including conversions of bioactive lipid metabolites such as linoleic acid derivatives. By integrating lipid-specific and accessory functions alongside vitamin biosynthesis linked to lipid metabolism, this resource enables a more holistic assessment of the interactions between lipids and their derivatives, the microbiome and their collective impact on human health.

In line with the need to expand lipid‑centric microbiome models, we examined how enterotype stratification shapes the distribution of lipid‑associated functions across the gut microbiome. We found that stratifying the BMIS dataset^21^ by enterotype explained the majority of the variance in lipid gene abundance. Notably, the two *Bacteroides* enterotypes remained those with the highest inferred lipolytic potential, in agreement with prior findings.^2^ ^,^ ^21^ Among these, *Bacteroides* 2 exhibited the highest number of unique lipid‑associated functions when normalized by total functional diversity. Interestingly, *Bacteroides* enterotypes have been associated with more Western-style diets, rich in protein and (saturated) fats.^44^ ^,^ ^45^ One contributing factor may be the relatively higher abundance of *Escherichia* species in the *Bacteroides* 2 enterotype,^21^ a species with a versatile lipolytic capacity (this study^21^). Alternatively, the enrichment of lipid‑associated functions in this enterotype may reflect a relatively greater contribution from small‑intestinal microbes compared with colonic inhabitants. This shift may be driven by the lower overall microbial load characteristic of this enterotype,^32^ ^,^ ^46^ which could increase the proportional representation of DNA originating from small‑intestinal communities in stool samples. Such a compositional bias may partially explain the distinctive lipid‑related functional repertoire observed for this enterotype.

Among the genes enriched in the *Bacteroides* 2 enterotype, steroidal metabolite sulfonation genes (*BtSULT, cysC*, and *cysD*) were particularly notable in the context of host health. These genes have been reported to modulate the sulfation of cholesterol and bile acids, with downstream effects on host immune regulation.^33^ We observed strong positive associations between sulfonation genes and serum bile acids, encompassing ursodeoxycholate (UDCA) as well as its isomeric, glycine‑ and taurine‑conjugated forms and their corresponding sulfated derivatives. These findings contrast with those of Yao et al.^33^, who reported no detectable sulfonation activity in *in vitro* incubations with unconjugated UDCA. This discrepancy may reflect the requirement for bile acid conjugation to enable sulfation, environmental factors influencing enzyme activity *in vivo,* or strain‑level variation in *BtSULT* substrate specificity. Notably, reduced host-mediated sulfation of bile acids has been linked to barrier dysfunction, inflammation, and carcinogenesis.^47^ Given that *Bacteroides* 2 is the enterotype most frequently associated with dysbiosis and the enterotype with enriched sulfonation genes,^46^ ^,^ ^48^ these findings raise the possibility that bacterial sulfonation activity may modulate, compete with, or perturb host sulfation pathways.^47^ Indeed, sulfonation genes have also been implicated in regulating host immune cell trafficking,^33^ providing a potential mechanistic link between microbial steroidal metabolism and host immune signalling in this enterotype.

Beyond bile acid modification, regional differences in lipid metabolism were also evident at the level of bacterial membrane biosynthesis. Genes most strongly enriched in the small intestine were associated with phospholipid biosynthesis (*fakA, fakB2*), facilitating the incorporation of fatty acids into bacterial membranes.^35^ While these lipid genes were detected across both intestinal regions, their enrichment in the small‑intestinal microbiome suggests a context‑specific adaptive advantage. Specifically, the incorporation of exogenous fatty acids into bacterial membranes is energetically more efficient than *de novo* fatty acid synthesis via reverse β‑oxidation.^49^ The metabolic energy saved by bypassing *de novo* synthesis can instead be allocated to other anabolic processes, thereby promoting faster growth. This provides a selective advantage in the small intestine, an environment characterized by rapid transit and strong competitive pressure for efficient resource utilisation.^50^ In addition to energetic efficiency, these genes may enhance tolerance to the fluctuating oxygen availability observed in the highly dynamic small‑intestinal milieu.^51^ Notably linoleic acid hydratase (*CLA-HY*) and linoleic acid (*CLA-ER*) exhibited similar patterns and correlations as the exogenous fatty acid incorporation genes, suggesting a potential functional relation.^36^ ^,^ ^52^ This gene class has been associated with increased bacterial stress tolerance suggesting a link with the membrane.^36^ Another lipid transformation gene more abundant in the small intestine was cholesterol dehydrogenase (*ismA*),^53^ ^,^ ^54^ which transforms cholesterol to cholestenone and can decrease the cholesterol level in the host. Collectively, these findings underscore the capacity of small intestine community to modify dietary lipids and their derivatives, with potential health consequences for the host.

In contrast to these anabolic and membrane‑associated pathways, we observed no strong enrichment of genes related to lipid degradation pathways, such as glycerol fermentation or β‑oxidation, in the small intestine, despite residual oxygen potentially supporting aerobic or microaerobic β‑oxidation.^26^ ^,^ ^27^ ^,^ ^51^ In the colon, strictly anaerobic conditions combined with limited or unfavorable electron acceptors render complete β‑oxidation both kinetically and energetically challenging.^26^ ^,^ ^27^ Under such conditions, only reverse β‑oxidation linked to chain elongation has been reported, particularly in the presence of linoleic acid.^55^ Consistent with this, β‑oxidation genes were primarily detected in opportunistic taxa such as *Escherichia* and *Klebsiella*, which are characteristic inhabitants of the small intestine.^50^ However, despite the potential energetic benefit of β‑oxidation in this region, these genes were only modestly enriched. Similarly, glycerol degradation genes were not strongly enriched in the small intestine. Collectively, these findings suggest that gut microbes preferentially exploit dietary lipids for anabolic processes, membrane remodeling, and host‑interactive transformations rather than serving as major degraders of dietary lipids.

Our study demonstrates that the small intestinal microbiome exhibits a markedly greater genetic potential for lipid metabolization compared to the colonic microbiome. This observation aligns with the physiological context that under a habitual dietary pattern, lipids are absorbed primarily in the small intestine and only a limited amount reach the colon,^6^ which likely explains the enrichment of lipid genes in the upper regions of the intestinal tract. Previous studies have reported shifts in fecal microbiome composition in participants consuming high-fat diets.^16^ ^,^ ^17^ ^,^ ^56^ However, these changes were most likely driven by reduced fiber intake rather than by the lipids themselves. Because the majority of lipid and their derivatives are rapidly absorbed in the small intestine, microbial transformation of these compounds would be expected to occur predominantly within this segment of the gastrointestinal tract when the relevant metabolic pathways are active. The short transit time, rapid host absorption, and relatively low microbial cell density impose constraints on microbial metabolic activity. Nevertheless, certain transformations, such as conjugated linoleic acid (CLA) formation have been shown to in occur quickly in specific microbial systems^57^ and can exert profound effects on host physiology. Mouse studies further underscore the importance of small intestinal microbiota in lipid derivative conversion.^19^ ^,^ ^58^ Yet, the small intestine represents a highly dynamic environment characterized by fluctuations in substrate availability, microbial load and composition, bile acid concentrations, pH, oxygen levels, and transit time.^6^ ^,^ ^42^ ^,^ ^59^ As our analysis is based on gene abundance and therefore reflects metabolic potential rather than direct activity, future studies integrating metagenomics with transcriptomic, proteomic, metabolomic, and functional approaches will be required to determine the extent to which these pathways are expressed and active *in vivo*. To gain clearer insights into how microbes transform lipids and their derivatives, and influence host health, well-designed *in vivo* trials focusing specifically on the small intestine are essential. Moreover, the small intestine is a highly dynamic environment, with oxygen availability, nutrient concentrations, microbial load, and microbial composition varying substantially along its length.^39^ ^,^ ^60^ This spatial heterogeneity contributes to considerable variability both within and between individuals, highlighting the importance of incorporating longitudinal sampling strategies in future studies to capture these dynamics over time.^42^

## Limitations

Our analysis considered both colonic as well as small intestine communities, however, we were limited in small intestine community datasets availability. The small intestine dataset used in our study was of participants with an ileostomy or ileoanal pouch, who although reported healthy, have a small intestine community which can differ from people without an ileostomy or ileoanal pouch. While stoma effluent represents the most effective method for studying the small intestine at large-scale and with high resolution, two important limitations must be acknowledged: the underlying disease history of the participants and the inability to capture physiological and microbial differences across different sections of the small intestine. Future studies using advanced sampling methods, such as endoscopic pills, may help expand and validate our findings in healthy individuals and further explore the diversity of microbial lipid metabolism across the small intestine. Although sampling, sequencing and bioinformatic processing was harmonized across cohorts, residual cohort-specific batch effects cannot be excluded. Because the overlap between location and cohort conventional batch-correction approaches could not be applied. However, small intestine microbial compositions in our cohort aligns with previously reported compositions, suggesting that any potential remaining batch effect should be minimal. This reflects the limited availability of high-quality metagenomes from regular small intestinal communities, obtained without ileostomy or ileoanal pouch, but by catheter or ingestible pills. Consequently the resolution at which their contributions to host health can be assessed remains limited. Another limitation was that for many of the genes we now assign functions to have not been functionally characterized in a gut context with biochemical assays or similar. Extensive evaluation of lipid gene function *in vitro* using gut-representing species and strains and/or heterologous expression platforms would be needed to strengthen the function assignments used. We base our findings on gene richness and abundance comparisons, while gene expression levels are likely more relevant to predict the lipid-modifying activity levels. The incorporation of meta-transcriptomic data, particularly from small intestinal samples collected following high‑fat consumption, would enable a more direct link to be established between specific microbial taxa, their functional genes, and *in situ* metabolic activity.

## Materials and methods

### Literature searches

To identify genes associated with microbial dietary lipid conversions literature searches were carried out until June 2024 using PubMed and Scopus databases and the Google Scholar search engine. The search strategy included terms related to microbial lipid metabolism (e.g., “beta oxidation”, “lipid conversion”, “fatty acid transportation”, “fatty acid degradation”, “choline degradation/transformation”) with gene-related keywords (e.g., “metabolism”, “gene expression”) and microbial taxa of interest (e.g., “*Lactobacillus*”, “gut microbiota”, “small intestine microbiota”). English articles were screened on relevance based on title and abstract, followed by a full-text review if the article was deemed relevant. Reference lists of key articles were manually searched to identify additional relevant studies. We focused on articles which described the use of techniques like (meta)genomics, (meta)transcriptomics combined with metabolomics to link the gene to a functionality. The translated amino acid sequences of genes were extracted from the publication which reported functional annotations or experimental validation of lipid conversion activity or extracted from UniProt, NCBI RefSeq or KEGG databases. The final gene list was curated based on reproducibility across studies and databases (Interpo, KEGG, UniProt). Finally, genes were grouped into functional categories representing shared metabolic pathways (e.g. β-oxidation) or associations with specific lipid classes (e.g. sterols).

### Construction of hidden Markov models

To enable sensitive detection of genes involved in microbial lipid conversions we constructed Hidden Markov Models (HMMs) based on the genes identified in our literature study. For all analyses presented in this study we used the translated amino acid sequences of the genes of interest. Orthologous groups of the predicted proteins of the reference genomes were constructed with OrthoAgogue (version 1.0.3)^61^ and a cut-off value of 1 * 10^−5^. Candidate orthologous groups corresponding to lipid genes described in the literature were identified and extracted for downstream analyses. MUSCLE^62^ (version v3.8.1551) was used to create multisequence alignment (MSA) files and all MSAs were manually inspected to assess alignment quality, including the presence of truncated sequences, poorly aligned proteins, and inconsistencies in conserved functional domains. HMMs were constructed from the curated MSAs using hmmbuild (HMMER version 3.4, www.hmmer.org) with default parameters. For each HMM a model-specific gathering threshold (GA score) was established through an automated procedure. The threshold was calculated as midpoint between the lowest-scoring target sequence (proteins belonging to the target orthologous group) and the highest-scoring target sequence (proteins from the reference genome set not belonging to the group). This automated approach was successful for the majority of the HMMs. For the remaining HMMs, GA scores were manually adjusted to optimize discrimination between positive and negative sequences, while allowing for a limited number of false-positive or false-negative classifications. The resulting GA score was embedded in each HMM file to guide sequence classification. This approach resulted in model-specific classification thresholds and is conceptually similar to the use of curated gathering thresholds in the Pfam database. To evaluate the functional consistency of the HMM seed sequences, all proteins used for model construction were annotated using InterProScan^30^ (version 5.73-104.0), and the resulting Pfam, NCBIfam, PANTHER, and HAMAP annotations were compared with the expected function of each protein (Supplemental Table S3). Annotations were classified as exact matches, family match or domain match, providing an independent assessment of the functional consistency of the reference dataset used for HMM development. Lipid genes in the datasets of interest were identified using hmmscan (HMMER version 3.4, www.hmmer.org) with the “--cut_ga” flag.

### Cohort and metadata description

We analyzed three independent, well-established cohorts to characterize the lipid-metabolizing potential of distinct gut microbial communities across intestinal regions (enterotypes; small intestine versus colon) and in the context of metabolic health (metabolic syndrome and prediabetes). The cohorts studied were: (1) Body Mass Index Spectrum (BMIS) cohort,^21^ a sub-cohort of the European MetaCardis study, comprising individuals with impaired glucose tolerance (prediabetes), metabolic syndrome with or without impaired glucose tolerance, and healthy controls (described in detail by Vieira-Silva et al.^21^); (2) Lifelines DEEP (LLD) cohort,^20^ a population-based cohort from the northern Netherlands; and (3) 1000IBD cohort,^22^ consisting of individuals with inflammatory bowel disease (IBD) recruited at the University Medical Center Groningen (UMCG).

From the LLD cohort, individuals with a complete set of data layers, including fecal metagenomic, fecal metabolomic, dietary, medical, and demographic data were selected to represent the general population colonic microbiome (*n* = 255). 56 individuals with an ileostomy or ileoanal pouch following total colectomy were selected from the 1000IBD cohort to represent the small intestinal microbiome, for whom the same data layers were available (for details, see the 1000IBD cohort paper.^22^) 819 individuals from the BMIS cohort were included to represent distinct colonic enterotypes in the context of health and metabolic disbalance. Serum metabolite data was available for the BMIS cohort instead of fecal metabolites.

### Sample collection and processing

For the BMIS cohort, fecal samples were collected following the *International Human Microbiome Standards* (IHMS) guidelines (modified SOP 04 V1; collection without anaerobic bag). In brief, study participants collected samples at home and immediately stored them at −20 °C until collection within 48 h. Samples were transported on dry ice and stored at −80 °C at the respective collection centers—Pitié-Salpêtrière Hospital (France), University Hospital of Leipzig (Germany), and Frederiksberg Hospital (Denmark).

Total fecal DNA was extracted in accordance with the IHMS guidelines (SOP 07 V2 H) and sequenced using the Ion Proton system (Thermo Fisher Scientific). Raw reads were cleaned using AlienTrimmer (v0.2.4) to remove residual sequencing adapters and trim low-quality nucleotides at the 3′ end. Cleaned reads were subsequently filtered to remove human and potential food contaminant DNA (using the *Homo sapiens* reference genome GRCh37-p10, *Bos taurus*, and *Arabidopsis thaliana*, applying an identity threshold of 97%). Microbial load was quantified by flow cytometry following previously described protocols (Vieira-Silva et al.^21^). Briefly, fecal aliquots were diluted, filtered, and stained with SYBR Green I, after which microbial cells were enumerated using a BD Accuri C6 flow cytometer with standardized staining and gating settings to calculate microbial load per gram of fecal material. Microbial genes were identified by mapping high-quality metagenomic reads to the 9.9-million-gene integrated human microbiome reference catalogue^63^ using Bowtie (version 2.2.6).

Plasma and serum samples were collected during a clinical examination visit following overnight fasting and stored at −80 °C at the respective centers. Serum samples were analyzed using a comprehensive metabolic phenotyping strategy combining in-house 1H-NMR spectroscopy, GC–MS, and targeted UPLC–MS/MS, with untargeted UPLC–MS data generated by Metabolon.

Further details on sample collection and processing are provided in Vieira-Silva et al.^21^, and Fromentin et al.^64^ For the LLD and 1000IBD cohorts, fecal samples were collected following the protocol described previously by Tigchelaar et al.^20^ and Imhann et al.^22^, respectively. In brief, participants were instructed to collect and immediately freeze their fecal samples at home at −20 °C. Samples were thereafter collected from participants' homes, transported on dry ice and stored at −80 °C in the laboratory. Microbial DNA was extracted using the Qiagen AllPrep DNA/RNA Mini Kit (Qiagen, cat. #80204) in combination with mechanical lysis and sent to the Broad Institute (Boston, MA, USA) for metagenomic shotgun sequencing (MGS) on the Illumina HiSeq platform. Metagenomic sequencing reads aligning to the human genome or Illumina adapters were removed using KneadData (version 0.4.6.1).

Fecal metabolomics profiling was performed by Metabolon (North Carolina, USA), detecting a total of 1684 metabolites. For more detail see Vich Vila et al.^65^

### Lipid gene detection and abundance estimation

To characterize the distribution of lipid genes across the human gut microbiome, our curated HMM library was applied to the protein sequences of 289,232 publicly available genomes from the Unified Human Gastrointestinal Genome (UHGG) database (v2.0.2). HMM searches were performed against the UHGP-90 non-redundant protein catalogue (downloaded from the EMBL-EBI MGnify FTP repository) using HMMER, with the curated gathering thresholds (--cut_ga) applied for gene detection. The resulting HMM hits were used to determine the presence and prevalence of lipid-metabolizing genes across genomes and species.

To identify our target lipid genes within the BMIS cohort, cleaned metagenomic reads from fecal samples were assembled into contigs using MegaHit (version 1.2.9). Protein-coding sequences (CDSs) were predicted from these contigs with Bakta (version 1.9.2) and subsequently scanned using our constructed HMMs to detect lipid genes. To estimate gene abundances, cleaned reads were aligned to all predicted CDSs with Bowtie2 (version 2.4.2), and CDS-level read counts were obtained with featureCounts (version 2.1.1). Read counts corresponding to HMM “hit” sequences were extracted and normalized by CDS length (in kilobases) and by the total number of cleaned reads (in millions), resulting in abundance estimates expressed as Reads Per Kilobase of transcript per Million mapped reads (RPKM).

For the LLD colonic and 1000IBD small intestinal cohorts, gene abundances were estimated using a MAG-based approach. Cleaned reads were assembled into metagenome-assembled genomes (MAGs), and protein-coding sequences (CDSs) were predicted with Bakta (version 1.8.1). These CDSs were scanned with our HMMs to identify lipid genes. Cleaned reads from each sample were then aligned to the pooled CDSs from all samples using Bowtie2 (version 2.5.4), and coverage was estimated with CoverM (version 0.7.0),^66^ correcting for CDS length (RPK). Abundance estimates for the identified lipid CDSs were further normalized by the total number of cleaned reads (RPKM). This MAG-based approach additionally enabled the assignment of lipid genes to specific bacterial species.

### Comparing lipolytic potential between gut communities (enterotypes)

Community typing (enterotyping) of the BMIS cohort genus-level microbial abundance profiles was previously performed by the Raes lab, as described in detail by Vieira-Silva et al.^21^ Four enterotypes were identified: *Bacteroides* 1 (Bact1), characterized by high relative abundances of *Bacteroides* and *Faecalibacterium*; *Bacteroides* 2 (Bact2), enriched in *Bacteroides* but depleted in *Faecalibacterium*; *Prevotella* (Prev), dominated by *Prevotella*; and *Ruminococcus* (Rum), characterized by low *Bacteroides* and *Prevotella* abundances.

To investigate differences in lipid-metabolizing potential between enterotypes, alpha diversity was assessed by comparing lipid gene richness (total unique lipid gene count) and relative lipid gene richness (lipid gene richness divided by total gene richness × 100) per metagenome. Total gene richness was calculated by counting the number of genes that were detected at least once in each sample, using the average number of genes counted in ten independent rarefaction experiments. Differences were established using Wilcoxon rank-sum test. Beta diversity was assessed by performing principal coordinate analysis (PCoA) on lipid gene abundances (restricted to genes present in ≥20% of samples) using Bray–Curtis distances. All subsequent analyses involving gene abundances were likewise limited to genes with a prevalence of at least 20% in the total cohort. Pairwise enterotype–enterotype differential abundance analyses were performed using linear regression (lm function in R) followed by Tukey's post-hoc tests (emmeans function in R, adjust = “tukey”), with multiple-testing correction applied using the false discovery rate (FDR).

Similarly, associations between metabolic health (PD, MBS, MBS-PD) and gut microbial lipid metabolism were explored by comparing total lipid gene richness, relative lipid gene richness, and individual lipid gene abundances against those of metabolically healthy controls. Differences in gene richness were assessed using the Wilcoxon rank-sum test, and differences in gene abundances were evaluated using linear regression, followed by FDR correction.

### Sequencing depth and lipid gene richness (supplementary analyses)

To assess whether sequencing depth (total number of cleaned metagenomic reads divided by microbial load) influenced detection of lipid genes, we performed Spearman's rank correlation between sequencing depth and lipid gene richness.

### Association between lipid genes and serum metabolites

Associations between microbial lipid gene abundances and serum lipid metabolites within the BMIS cohort were assessed using the bioBakery tool HAIIA (Hierarchical All-against-All Association Testing) (version 0.8.40).^37^ The analysis was performed on lipid genes detected in at least 20% of samples. Multiple testing corrections were applied using FDR.

### Microbial lipid metabolism and host health parameters

Redundancy analysis (RDA) was performed in R using the RLdbRDA function developed by the RaesLab (https://github.com/raeslab/RLdbRDA) to assess associations between host phenotypes and lipid gene abundance profiles within the LLD cohort. Host variables included age, sex, BMI, macronutrient intake, and medication use. Categorical variables were only included if each category contained at least 10 samples. Individual lipid gene abundances were correlated with diet quality and gut health using linear regression. Diet quality was derived from food frequency questionnaires and energy-adjusted to grams per 1000 kcal. Participants were assigned scores from 0–4 based on quintiles of intake, with higher scores for favorable foods (e.g., vegetables, fruit, whole grains, legumes/nuts, fish, oils, unsweetened dairy, coffee, tea) and reversed scores for unfavorable foods (e.g., red/processed meat, butter, sugar-sweetened beverages). The total dietary quality score was the sum of all food group scores (range 0–48). Gut health was assessed using the gut microbiome wellness index 2.^38^

### Lipid metabolism in the small intestine

To assess the lipolytic potential of the small intestinal microbiome relative to that of the colon, we compared fecal samples from the general population (LLD cohort), representative of colonic communities, with fecal samples from individuals with an ileostomy or ileoanal pouch (1000IBD cohort), representative of the small intestine. Differential analyses of lipid gene richness, β-diversity, and gene abundances were performed as described above. Total gene richness per sample was calculated as the number of unique coding sequences with an estimated CoverM (version 0.7.0)^66^ coverage greater than 0.1×.

### Genus-level estimation of lipid gene abundances in the small intestine versus colon

To determine the taxonomic origin of lipid genes in the small intestine (1000IBD ileostomy and ileoanal pouch samples) versus colon (LLD fecal samples), total lipid gene abundances were aggregated at the genus level based on the taxonomic annotation of each corresponding CDS derived from MAGs.

## Supplementary Material

Supplementary material Supplemental Material Mapping the genetic landscape of microbial lipid transformation in the intestinal microbiome.docx

Mapping the genetic landscape of microbial lipid transformation in the intestinal microbiome_SupplementaryTables.xlsx Mapping the genetic landscape of microbial lipid transformation in the intestinal microbiome_SupplementaryTables.xlsx

## Data Availability

The datasets analyzed in this study are available through controlled-access repositories. The LifeLines-DEEP^20^ metagenomic sequencing data are available through the European Genome-phenome Archive (EGA) under study accession EGAS00001001704 and dataset accession EGAD00001001991. The MetaCardis^21^ dataset is available through the EMBL-EBI European Nucleotide Archive (ENA) under accession PRJEB37249. The 1000IBD^22^ multi-omics dataset is available through the EGA under study accession EGAS00001002702. Access to human participant data may require approval from the respective Data Access Committees and must comply with the relevant ethical and legal requirements. The hidden Markov models (HMMs) developed for this study, which target gut microbial lipid metabolizing functions, are publicly available at the following DOI: https://doi.org/10.5281/zenodo.20795554
