# Supplementary material for: Mapping the genetic landscape of microbial lipid transformation in the intestinal microbiome
Source: Gut Microbes. 2026 Nov 11;18(1):2756735. doi: 10.1080/19490976.2026.2756735 (PMC13761379; doi:10.1080/19490976.2026.2756735)
Supplement: Supplementary file 1 [file KGMI_A_2756735_SM9818.docx]

Supplemental Material - Mapping the genetic landscape of microbial lipid transformation in the intestinal microbiome

Authors: Laura C. Valk^1†^, Renate A.A.A. Ruigrok^2,3^, Harro Timmerman^1^, Johannes R. Björk^2,3^, Qiyan Li^2,3^ Shuyan Zhang^2,3^, Rui Menses^4,5^, Jorge Francisco Vázquez Castellanos^4,5^, Nicole de Wit^1^, Jeroen Raes^4,5^, Rinse K. Weersma^2,3^, Michiel Kleerebezem^6^, Arnau Vich-Vila^4,5^, Jos Boekhorst^6^

1) Wageningen Food and Biobased Research, Wageningen University and Research, Wageningen, The Netherlands
2) Department of Gastroenterology and Hepatology, University of Groningen, University Medical Center Groningen, Groningen the Netherlands.
3) Department of Genetics, University of Groningen, University Medical Center Groningen, Groningen the Netherland
4) Laboratory of Molecular Bacteriology, Department of Microbiology and Immunology, Rega Institute, KU Leuven, Leuven, Belgium
5) Center for Microbiology, VIB, Leuven, Belgium
6) Host-Microbe Interactomics Group, Animal Sciences Department, Wageningen University & Research, Wageningen, The Netherlands

† corresponding author

**Figure S1 | Functional congruency for the 88 seed proteins.** InterProScan annotations were used to assess the functional support for each HMM seed protein. Support was classified as exact functional assignment (green), protein family-level support (purple), or domain-level support (yellow). Gene labels are colored according to their associated functional group.

**Figure S2 | Distribution and conservation of lipid genes across UHGG genomes.** The conservation of bacterial lipid metabolism genes varied markedly across microbial taxa present in the human gut. A) Distribution of lipid metabolism genes classified as core or accessory within each genus. Core genes were defined as being present in ≥ 90 % of genomes within a species, whereas accessory genes were present in <90% of genomes. Colour indicates gene conservation status, with blue-green representing core genes (≥ 90 % prevalence) and red representing accessory genes (<90% prevalence). B) Dot plot showing the prevalence of each lipid-metabolising gene across species (x-axis) and the proportion of species in which the gene is part of the core genome (y-axis). C) Hierarchical clustering of genes based on their presence/absence across bacterial genomes (n = 289,232), using Jaccard distance calculated from binary gene occurrence profiles and average-linkage (UPGMA) clustering. Branch height represents the Jaccard dissimilarity between gene occurrence profiles, with lower branch heights indicating more similar genome co-occurrence patterns. The colour bar denotes the annotated metabolic pathway of each gene.

**Figure S3 | Permutation analysis of the different parameters influencing lipid gene variation per sample**. Enterotype was the highest explanatory value for lipid gene variation in the MetaCardis dataset^1^. Top 50 variables according to R^2^ are shown here. Significant variables (p-value < 0.05) are highlighted with an asterisk.

**Figure S4 | No significant relationship between total gene richness and sequencing depth per enterotype was identified**. (A) The sequencing depth (Total reads / Microbial Load (g)) was determined per sample and grouped per enterotype. (B) The sequencing depth was correlated to the total lipid gene richness (number of unique lipid genes per sample). The significance between sequencing depth and total lipid gene richness per enterotype was determined with the Wilcoxon rank-sum test. The samples are coloured according to their enterotype; Yellow = Ruminococcus, Blue = Bacteroides 1, Red = Bacteroides 2 and Green = Prevotella.

**Figure S5 | Lipid gene abundance associations with serum lipid levels (MetaCardis-BMIS).** Significant results of the hierarchical All-against-All association testing (HAllA^2^) between microbial lipid gene abundances per metagenome and serum lipid metabolites. Red indicates positive associations and blue negative associations. Colour intensity reflects the strength of the association (Spearman’s rho). Lipid genes are coloured according to their functional group. Numbers indicate distinct association clusters.

**Figure S6 | Enterotype-driven clustering and health group stratification in lipid gene abundance in MetaCardis-BMIS cohort.** Principle coordinate analysis based on Bray-Curtis distances calculated from the relative abundances of lipid metabolization genes in the MetaCardis-BMIS cohort. Each dot represents one sample (metagenome), colored by enterotype: yellow for Ruminococcus (Rum), blue for Bacteroides 1 (Bac 1), red for Bacteroides 2 (Bac 2) and green for Prevotella (Prev). Arrows were arrows enlarged by factor of 18 and indicate the 10 lipid genes with the highest loadings and stratification group, showing their contribution to variation along the first two PCo axes. Ellipses are 2D approximations at a confidence level of 90% and the outlined circles are the centroids of the ellipses. Lipid genes are colored according to their functional group.

**Figure S7 | Lipid gene abundance associations with faecal metabolite levels (LifeLines DEEP (LLD)).** Significant results of the hierarchical All-against-All association testing (HAllA)^2^ between microbial lipid gene abundances per metagenome and faecal metabolites. Red indicates positive associations and blue negative associations. Colour intensity reflects the strength of the association (Spearman’s rho). Numbers indicate distinct association clusters.

**Figure S8 | Lipid gene abundance differences between Diet Score groups and Gut Microbiome Wellness 2 index score (LifeLines DEEP (LLD)) shows little difference** Differential abundance of lipid genes detected in >20% of samples between different (A) Diet Scores, (B) Gut Microbiome Wellness Index 2 (GMWI2) Score^3^, assessed using linear regression followed by Tukey’s post-hoc test. Red indicates enrichment, blue underrepresentation. Estimate refers to the model coefficient representing the difference in outcome between Diet Score and Gut Microbiome Wellness Index 2 Score, respectively. Significance: *FDR < 0.05, **FDR < 0.01, ***FDR < 0.001. (B)

**Figure S9 | Distance based redundancy analysis with host phenotype and lipid genes of the LifeLines DEEP dataset**^4^**.** Significant associations were only observed at low FDR threshold (FDR = 0.1). The analysis was performed using the RLdbRDA tool (available at <https://github.com/raeslab/RLdbRDA>).

**References**

1.	Vieira-Silva S, Falony G, Belda E, Nielsen T, Aron-Wisnewsky J, Chakaroun R, Forslund SK, Assmann K, Valles-Colomer M, Nguyen TTD, et al. Statin therapy is associated with lower prevalence of gut microbiota dysbiosis. Nature 2020; 581:310–5.

2.	Ghazi AR, Sucipto K, Rahnavard A, Franzosa EA, McIver LJ, Lloyd-Price J, Schwager E, Weingart G, Moon YS, Morgan XC, et al. High-sensitivity pattern discovery in large, paired multiomic datasets. Bioinformatics 2022; 38:i378–85.

3.	Chang D, Gupta VK, Hur B, Cobo-López S, Cunningham KY, Han NS, Lee I, Kronzer VL, Teigen LM, Karnatovskaia LV, et al. Gut Microbiome Wellness Index 2 enhances health status prediction from gut microbiome taxonomic profiles. Nat Commun 2024; 15:7447.

4.	Tigchelaar EF, Zhernakova A, Dekens JAM, Hermes G, Baranska A, Mujagic Z, Swertz MA, Muñoz AM, Deelen P, Cénit MC, et al. Cohort profile: LifeLines DEEP, a prospective, general population cohort study in the northern Netherlands: study design and baseline characteristics. BMJ Open 2015; 5:e006772.
